# Upper Airway Dimensions among Different Skeletal Malocclusions: A Retrospective Observational Study by Cephalometric Analysis

**DOI:** 10.3390/dj12010012

**Published:** 2024-01-03

**Authors:** Maria Francesca Sfondrini, Simone Gallo, Maurizio Pascadopoli, Paola Gandini, Caterina Roncoroni, Andrea Scribante

**Affiliations:** Unit of Orthodontics and Pediatric Dentistry, Section of Dentistry, Department of Clinical, Surgical, Diagnostic and Pediatric Sciences, University of Pavia, 27100 Pavia, Italy

**Keywords:** orthodontics, otorhinolaryngologic diseases, cephalometry, malocclusion, angle classes, Caucasian

## Abstract

The aim of the present work was to investigate the upper airway dimensions in adult non-orthodontic patients, equally divided according to their skeletal class. Methods: In this retrospective cross-sectional study, lateral cephalometric radiographs of adult patients referred for orthodontic consultation were collected. Cephalometric tracing was performed with dedicated software. For each measure, descriptive statistics were calculated. Cephalometric measurements between the different skeletal classes were compared. Linear regressions were performed between upper airway diameters and cephalometric measurements, sex and age. Significance was predetermined for *p* < 0.05. Results: Lateral cephalometric radiographs of 120 patients were reviewed. Nasopharynx length (NL) and depth (PD) measurements were significantly shorter in skeletal class III patients (*p* < 0.05). The superior pharyngeal airway space (SPAS) was found to be significantly shorter in class III patients as compared to class II patients (*p* < 0.05), and the mean airway space (MAS) of class I patients was found to be significantly shorter compared to class II patients (*p* < 0.05). Palate length (PL) values were found to be significantly longer in class I (*p* < 0.05). Linear regressions showed that the sella-nasion-A point angle (SNA) and Riedel’s angle between point A, the nasion and point B (ANB) significantly influenced NL and PD (*p* < 0.05). Conclusions: Class III patients show significantly shorter nasopharynx measurements; clinicians should consider that this sagittal discrepancy could be related to an altered anatomy of the upper respiratory tract.

## 1. Introduction

The evaluation of the upper airway is an issue of notable concern for orthodontists. Various studies have assessed the correlations between upper airway cephalometric measurements and craniofacial and occlusal patterns [1,2,3]. Lateral cephalometric radiographs are still widely performed in dental practices for orthodontic diagnosis [4]. Despite the diffusion of cone beam computed tomography (CBCT) and its reliability [5], cephalometric analysis is still part of the routine initial diagnostic records necessary for the planning of orthodontic treatment and can also be used for upper airway analysis [6]. It is commonly recognised that a bidirectional relationship occurs between a correct occlusion and the proper development and functionality of the upper airways based on Moss’s principle according to which “form follows function” [7].

A study conducted by Di Carlo et al. [8] aimed to assess through a 3D radiological evaluation if the upper airway’s morphology and dimensions vary among people reporting various craniofacial features. Ninety young adult patients without respiratory problems and no rhino-surgery performed were recruited, among which thirty were categorised as Class I (−0.5 < ANB < 4.5), thirty as Class II (ANB > 4.5) and thirty as Class III (ANB < −0.5). The supine position was considered in each patient to perform the cone-beam computed tomography (CBCT) scans. Cephalometric landmarks were detected in 3D. A correlation was conducted considering the dimensions on the sagittal and transversal planes, cross sections and partial and total volumes of the upper airway. This correlation was conducted with the cephalometric measurements in all three planes. Moreover, the upper airway cross-sectional minimal area was also studied. Relationships between upper airways’ dimensions and morphologies and skeletal malocclusion were not found to be significant. Craniofacial morphological differences were not correlated with the upper airway volumes’ variations. A clinically significant relation was found between the minimal area and the total upper airway volume.

Various authors have investigated the possible differences in airway dimensions in specific conditions like syndromic patients [9], mandibular traumas [10] and obstructive sleep apnoea syndrome (OSAS), the latter being one of the major areas of research in the last years addressing upper airway analysis [11]. Additionally, the morphological variations of upper airways have been also investigated in relation to various treatments such as those involving the use of functional appliances [12,13], orthodontic therapy [14,15] and orthognathic surgery [16,17]. Bucci and colleagues [18] conducted a systematic review regarding the effect of maxillary expansion on the upper airways’ features. The quality of most of the included reviews ranged from low to critically low, with only one systematic review rated as high quality. Therefore, according to the results of the study, a statistically significant increment in nasal linear dimensions was reported in the short as well as in the long term supported by low-/critically low-quality systematic reviews, whereas the significant increase in nasal cavity volume was the exclusive outcome supported by a high-quality systematic review. It is important to notice that very few or no follow-up studies after rapid palatal expansion have been published. 

Besides the changes caused by orthodontic appliances, to the best of our knowledge, a cephalometric assessment of upper airways in adult Caucasian subjects who did not previously receive any kind of orthodontic, orthopaedic, or surgical treatment has not been conducted. Accordingly, the goal of the present study was to investigate the baseline upper airway dimensions in adult patients belonging to the three different skeletal classes. The statistical null hypothesis corresponded to no differences in cephalometric measurements between the three angle classes. 

## 2. Materials and Methods

### 2.1. Study Design, Setting, Participants, Variables

This was a retrospective observational cross-sectional study approved by the Unit Internal Review Board (approval n°: 2022-0209). The study was registered on clinicaltrials.gov (registration number: NCT05725980). STROBE guidelines for observational studies were considered for reporting the present research.

Lateral cephalometric radiographs of adult orthodontic patients referred for orthodontic consultation from 2021 to 2023 were screened from February to March 2023. The selection was conducted based on the following inclusion criteria: age between 18 and 50 years old; permanent dentition; Caucasian patients; lateral cephalometric digital radiograph executed in the natural head position (with teeth in contact and lips relaxed) for orthodontic reasons. The following exclusion criteria were also considered: previous orthopaedic/orthodontic treatments; orthognathic surgery; maxillofacial traumas and nasopharyngeal pathologies. 

Radiographs were performed as part of the diagnostic process, thus ensuring that this study abides by good clinical practice.

For each lateral cephalometric radiograph, cephalometric tracing was performed with dedicated software (DeltaDent, version 2.2.1, Outside Format, Spino d’Adda, Italy). 

Riedel’s ANB angle [19] was adopted to determine the skeletal class of patients as follows:

- ANB = 2° ± 2° → Class I,
- ANB > 4° → Class II,
- ANB < 0° → Class III.

Steiner’s SN^GoGn angle [20] was used to evaluate skeletal divergence:

- SN-GoGn = 32° ± 5 → normodivergence,
- SN-GoGn < 27° → hypodivergence,
- SN-GoGn > 37° → hyperdivergence.

The following parameters were used to evaluate biprotrusion:

- SNA > 84° and SNB > 82°.

The following parameters were used to evaluate biretrusion:

- SNA < 80° and SNB > 78°.

The list of the anatomic landmarks is shown in Table 1. For the upper airway analysis, points and measurements shown in Table 2 were considered.

**Figure 1:**
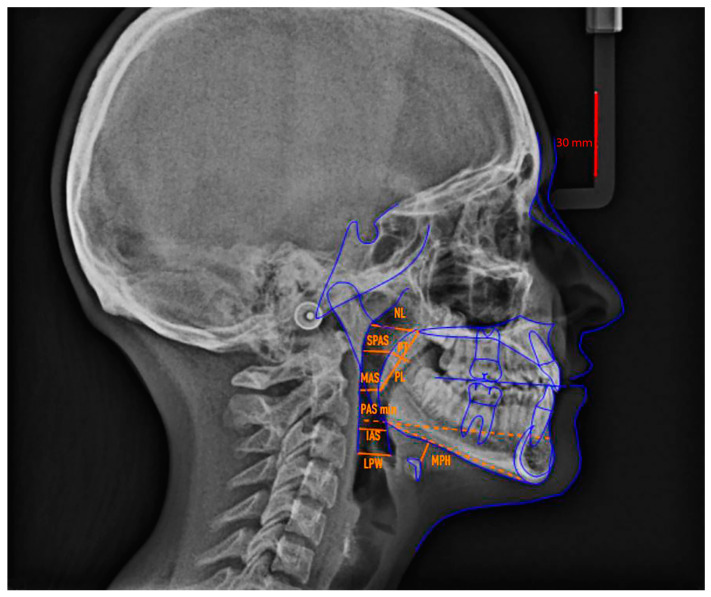
Anatomic landmark used for the upper airways cephalometric analysis.

Figure 1 shows the cephalometric tracing with upper airway analysis.

### 2.2. Data Measurement and Bias

The cephalometric tracings were performed by two calibrated operators. Intra-rater reliability was assessed by re-doing 20% of the cephalometric analyses after 2 weeks; therefore, 24 lateral cephalometric radiographs underwent double analysis. Intraclass correlation coefficients (ICC) of 0.93 and 0.92 were obtained for the two operators, representing excellent strength of agreement. Inter-rater reliability was assessed by comparing the same cephalometric tracings, obtaining a 0.91 coefficient of agreement.

### 2.3. Study Size

Sample size calculation was performed considering the “soft palate length (PL)” as the primary outcome. Considering alpha = 0.05 and power = 95%, as well as previous literature, an expected mean value of 31.05 with a standard deviation of 4.16 was hypothesised, with an expected mean difference of 3.34 [21]. In total, 40 records for each of the three groups (Class I, Class II and Class III malocclusions) were required. 

### 2.4. Statistical Methods 

For each cephalometric measurement belonging to the three skeletal classes, descriptive statistics (mean and standard deviation) were calculated. The Kolmogorov–Smirnov test was used for assessing data normality. The comparisons among skeletal classes were not normally distributed; therefore, Kruskal–Wallis analysis followed by Dunn’s post hoc test was performed. The comparisons for protrusion and divergence were normally distributed; therefore, ANOVA followed by Tukey’s post hoc tests was performed. Linear regressions were performed between upper airway diameters, SNA, SNB, ANB, SN^GoGn, ANSPNS^GoGn, PT, PL, sex and age. Significance was predetermined at *p* < 0.05. 

R^®^ software (version 3.1.3 R Development Core Team, R Foundation for Statistical Computing, Wein, Austria) was used to conduct the statistical analysis.

## 3. Results

Regarding the sample size calculation, the records of 40 patients per group were reviewed and divided into three skeletal classes, with a total of 120 patients’ records (71 F and 49 M). The baseline data of participants in each group are shown in Table 3. 

The results of the comparison of cephalometric outcomes among the skeletal classes, position of the jaws and skeletal divergency are shown in Table 4. Nasopharynx measurements NL and PD were found to be significantly lower in skeletal class III patients as compared to class I and II patients (*p* < 0.05). Significant differences were observed between biretruded and normoposition as well as between biprotruded and normoposition for PD and IAS values (*p* < 0.05). Instead, no significant differences were found in general comparing hypodivergent, normodivergent and hyperdivergent patients (*p* > 0.05); the latter group was typically associated with respiratory and muscular difficulties in hyperdivergent patients. 

Linear regressions (Table 5) were found to be significant for SNA and ANB as predictors and nasopharynx measurements (NL and PD) as dependent variables (*p* < 0.05). The results of the linear regression revealed a significant relation between the PL predictor and SPAS (*p* < 0.05). MAS, IAS and PAS min were found to be influenced by sex (*p* < 0.05), with higher values for males and lower ones for females, while only MAS and PAS min were influenced by age (*p* < 0.05), with lower values for older patients and higher values for younger patients. The LPW measure was found to be influenced by the MPH predictor; a significant linear regression was also found for PL with the PT predictor (*p* < 0.05).

## 4. Discussion

The upper airway is a complex structure whose functions consist of respiration, deglutition and phonation. The bidirectional relationship between breathing and facial growth has been extensively demonstrated; the impact of the mode of breathing and head posture on the facial growth pattern was described in the ‘soft-tissue stretching hypothesis’ by Solow and Kreiborg [22]. Harvold described a variety of skeletal, dental and muscular alterations in animals with artificially obstructed nasal airways [23]. Warren and Spalding, conversely, revealed that the relationship between nasorespiratory function and dentofacial development is controversial [24].

To date, many studies have investigated the possible differences in airway dimensions under specific conditions, particularly in obstructive sleep apnoea syndrome (OSAS) [11,12]. Moreover, the morphological changes of upper airways have been also investigated in relation to various treatments [13,14,15,16,17,18]. In the present study, the aim was to consider a thorough bidimensional analysis of the upper airway in Caucasian adult patients who did not receive any previous orthopaedic/orthodontic/surgical treatment, clustered by the skeletal class, divergence and protrusion of the jaws Measurements from previous studies were included to perform a thorough evaluation [21,25,26].

The statistical null hypothesis of the study has been rejected in part. In fact, nasopharynx measurements NL and PD were found to be significantly lower in skeletal class III patients as compared to class I and II patients (*p* < 0.05). Significant differences were found for protrusion values in PD and IAS evaluation (*p* < 0.05). Similarly, no significant differences were found in general comparing hypodivergent, normodivergent and hyperdivergent patients (*p* > 0.05).

Table 6 shows the reference values for the outcome tested in the study.

On the basis of the results of the present study, the most relevant evidence obtained is the fact that nasopharynx measurements were significantly reduced in skeletal III class patients, which could be due to maxillary contraction. This finding is consistent with previous research in which a volume increase in the nasal cavity and nasopharynx was observed after maxillary expansion. Li et al. [27] aimed to assess dimensional and volumetric changes of the upper airways before and after rapid maxillary expansion with mini-implants insertion (MARME) and to find correlations between the changes of the upper airways and the vertical skeletal pattern in young adults. According to their findings, MARME caused a volume increase in the nasal cavity and nasopharynx, encompassing an expansion of the nasal osseous and maxillary width. Similarly, Chang and his colleagues [28] assessed the upper airways’ dimensional variations in patients reporting maxillary constriction treated by rapid maxillary expansion using a 3D evaluation by means of CBCT. The authors noted that the cross-sectional airway from the posterior nasal spine to the basion showed a significant augmentation of 99.4 mm (+59.6%), thus confirming the clinical benefit of rapid maxillary expansion of the maxilla as suggested by other authors [29,30].

According to the results of the current study, it is interesting to note that, besides finding significantly lower nasopharynx measurements in class III patients, the results of the linear regression revealed a significant relation for SNA and ANB as predictors and nasopharynx measurements (NL and PD) as dependent variables (*p* < 0.05). Therefore, the decrease in the nasopharynx measurements is directly proportional to the degree of maxillary sagittal dimensional reduction. Even though recent studies have found some modifications in the upper airway volume after orthopaedic treatment [13,31], airway pathologies should be dealt with using a multidisciplinary approach, involving an otolaryngologist in the diagnostic and therapeutic process.

A previous study has shown a significant correlation between maxillomandibular discrepancy and the severity of OSA [32]. Moreover, the reduction of nasopharyngeal width was correlated with a maxillomandibular hyperdivergent growth pattern. Such results support the presence of a correlation between sleep-disordered breathing and craniofacial features even if the cause–effect relation is still unclear. Based on this evidence, the authors suggest the importance of orthodontic evaluation in the management of paediatric OSA.

The main limitation of the current report could be the fact that the evaluation of airway morphology was conducted on the basis of cephalometry, i.e., by means of bidimensional analysis, as compared to other recent studies, like the ones mentioned above, which were based on a 3D analysis with CBCT. Even though three-dimensional evaluations could be more precise for the morphological evaluation of airways [33], it is important to take into careful consideration the higher biological risk deriving from this type of radiological assessment, following current guidelines [34]. Moreover, the observational nature of this report could be a limit considering the intrinsic limitations of this kind of study.

Based on these considerations, future perspectives should be considered. It could be interesting to further reproduce this analysis not only considering sagittal discrepancies but also other types of malocclusions as well as functional alterations. Every effort should be done to deepen the knowledge of the alteration of the airways in order to early detect patients with potential airway dysfunctionality related to maxillofacial morphology.

## 5. Conclusions

The upper airway is a complex structure, and the bidirectional relationship between breathing and facial growth has been recognised. Class III patients show significantly lower values of the nasopharynx, possibly related to the reduction of maxillary sagittal diameters, which is reflected in the upper airway dimensions. Clinicians should be aware of this evidence and should consider that this sagittal discrepancy could be related to an altered anatomy of the upper respiratory tract. Moreover, people should be instructed and motivated to make growing patients undergo an orthodontic evaluation in order to early detect skeletal and dental malposition, thus avoiding further problems not only related to the oral cavity.

## Figures and Tables

**Table 1:** Standard anatomic landmarks and planes for the cephalometric tracing.

Points and Measurements (Abbreviation)	Definition
Nasion (N)	Most anterior point on the frontonasal suture in the midsagittal plane
Sella (S)	Centre of the pituitary fossa of the sphenoid bone
Point A (A)	Deepest point of the curve of the anterior border of the maxilla
Point B (B)	Most posterior point in the concavity along the anterior border of the symphysis
Gonion (Go)	Point along the angle of the mandible, midway between the lower border of the mandible and the posterior border of the ascending ramus
Gnathion (Gn)	Most anterior and inferior points of the chin between the pogonion and menton
Basion (Ba)	Most anterior point of the foramen magnum
Anterior nasal spine (ANS)	Anterior tip of the sharp bony process of the maxilla at the lower margin of the anterior nasal aperture
Posterior nasal spine (PNS)	Posterior limit of the palatine bone
SNA	Angle between the sella, nasion and A point
SNB	Angle between the sella, nasion and B point
ANB	Angle between point A, the nasion and point B
SN	Plane between the sella and nasion
Sna-Snp	Bispinal plane
GoGn	Mandibular plane

**Table 2:** Anatomic landmarks and planes related to upper airway analysis.

Points and Measurements (Abbreviations)	Definition
*Soft palate*	
MPP	Middle point of the posterior wall of the soft palate
MPA	Middle point of the anterior wall of the soft palate
PT	Soft palate thickness
PL	Soft palate length
*Nasopharynx*	
Ad1	Point at the intersection between the posterior pharyngeal wall and PNS-Ba line
NL (nasopharynx length)	Distance between Ad1 and PNS
PD (nasopharynx depth)	Line parallel to the bispinal plane and connecting PNS and the posterior margin of pharynx
*Oropharynx*	
SPAS (point)	Projection of the MPP point on the pharyngeal wall
SPAS	Superior Pharyngeal Airway Space: from half of the posterior border of the soft palate (MMP) perpendicular to the closest point on the posterior wall of the pharynx
U1	Terminal part of the uvula
U2	Projection of the U1 point on the pharyngeal posterior wall
MAS	Mean Airway Space: U1—U2
T1	Intersection of the tongue’s base and the line of point B and Go
T2	T1 projection on the posterior wall of the pharynx along the parallel line Go-B
PAS min	Pharyngeal Airway Space minimum: T1—T2 distance along the Go-B parallel line
Ph1	Intersection of the tongue’s base and vallecula of epiglottis
Ph2	Ph1 projection on the posterior wall of the pharynx
IAS	Inferior Airway Space: from the superior margin of the epiglottis (Ph1) to the closest perpendicular point on the posterior wall of the pharynx
*Hypopharynx*	
Va1	Base of the epiglottic vallecula
Va2	Va1 projection on the posterior wall of pharynx
LPW	Lateral Pharyngeal Wall: distance between the epiglottic vallecula (Va1) and the posterior wall of pharynx perpendicular to Va1
*Hyoid bone*	
H1	The most cranial point of the body of the hyoid bone
H2	Projection of the H1 point on the perpendicular line of the inferior margin of the jaw (Go—Gn)
MPH	Mandibular Plane—Hyoid bone: distance from the most anterior and superior point of the hyoid bone (H1) perpendicular to the mandibular plane

**Table 3:** Demographic characteristics of the study sample. N is the number of patients; age is expressed as the mean ± SD.

	Angle Class I		Angle Class II		Angle Class III	
	*n*	Age	*n*	Age	*n*	Age
Female	27	27.86 ± 8.29	31	27.05 ± 7.59	13	28.36 ± 9.47
Male	13	27.68 ± 8.03	9	27.29 ± 8.09	27	28.38 ± 9.18

**Table 4:** Mean values and standard deviation (SD) of the upper airway measurements for the different skeletal classes and results of Dunn’s post hoc test. Mean values and standard deviation (SD) of upper airway measurements in biretruded and biprotruded patients and the results of Tukey’s post hoc test. Mean values and standard deviation (SD) of the upper airway measurements for different divergency patterns and Tukey’s post hoc test results. Significant differences were not observed between values with the same superscript. The same superscript letters indicate no significant differences (*p* > 0.05).

	Class I	Class II	Class III	Biretruded (*n* = 24)	Normoposition (*n* = 70)	Biprotruded (*n* = 26)	Hypodivergent (*n* = 24)	Normodivergent (*n* = 70)	Hyperdivergent (*n* = 26)
NL	19.88 ± 3.30 ^A^	21.07 ± 3.49 ^A^	17.26 ± 3.37 ^B^	18.19 ± 4.17 ^A^	18.62 ± 3.86 ^A^	27.3 ± 37.29 ^A^	19.82 ± 3.23 ^A^	22.36 ± 23.92 ^A^	18.55 ± 3.66 ^A^
PD	21.06 ± 3.22 ^A^	21.97 ± 3.45 ^A^	18.41 ± 3.45 ^B^	18.86 ± 3.95 ^A^	19.73 ± 3.59 ^A^	21.08 ± 3.1 ^A^	20.96 ± 3.16 ^A^	20.55 ± 3.77 ^A^	19.72 ± 3.86 ^A^
SPAS	11.95 ± 2.36 ^A^	12.57 ± 2.96 ^A^	11.25 ± 3.07 ^A^	11.26 ± 1.8 ^A^	11.78 ± 2.94 ^A^	12.27 ± 2.58 ^A^	11.60 ± 2.49 ^A^	11.88 ± 2.73 ^A^	12.04 ± 2.89 ^A^
MAS	8.99 ± 2.37 ^A^	10.19 ± 2.98 ^A^	10.08 ± 3.06 ^A^	8.9 ± 2.07 ^A^	9.64 ± 2.86 ^A^	10.04 ± 2.72 ^A^	10.13 ± 2.4 ^A^	9.51 ± 2.70 ^A^	9.65 ± 2.67 ^A^
PAS min	11.11 ± 2.85 ^A^	11.41 ± 3.40 ^A^	11.19 ± 3.52 ^A^	9.64 ± 2.24 ^A^	11.07 ± 3.25 ^A,B^	11.97 ± 3.2 ^B^	11.90 ± 2.76 ^A^	10.81 ± 3.19 ^A^	11.33 ± 3.12 ^A^
IAS	10.73 ± 2.51 ^A^	10.97 ± 3.39 ^A^	10.30 ± 3.33 ^A^	9.31 ± 2.72 ^A^	10.53 ± 3.17 ^A^	11.32 ± 2.62 ^A^	11.41 ± 2.7 ^A^	10.44 ± 3.12 ^A^	10.02 ± 2.51 ^A^
LPW	14.02 ± 3.24 ^A^	13.89 ± 3.02 ^A^	12.58 ± 3.75 ^A^	12.5 ± 3.44 ^A^	13.20 ± 3.65 ^A^	13.98 ± 3.19 ^A^	14.43 ± 2.81 ^A^	13.23 ± 3.50 ^A^	12.89 ± 3.16 ^A^
PL	33.29 ± 4.33 ^A^	30.93 ± 5.43 ^A^	30.85 ± 5.80 ^A^	29.4 ± 4.74 ^A^	32.22 ± 6.08 ^A^	32.21 ± 4.6 ^A^	32.17 ± 4.76 ^A^	32.23 ± 5.66 ^A^	30.20 ± 4.15 ^A^
PT	9.38 ± 1.64 ^A^	9.37 ± 2.61 ^A^	9.71 ± 2.51 ^A^	8.54 ± 1.83 ^A^	9.37 ± 2.25 ^A,B^	10.19 ± 1.74 ^B^	10.26 ± 1.74 ^A^	9.71 ± 2.51 ^A^	9.21 ± 1.99 ^A^
MPH	12.81 ± 4.72 ^A^	13.18 ± 5.30 ^A^	14.64 ± 4.17 ^A^	12.09 ± 4.94 ^A^	14.20 ± 4.00 ^A^	13.71 ± 4.15 ^A^	13.98 ± 3.84 ^A^	13.65 ± 4.77 ^A^	12.66 ± 5.40 ^A^

**Table 5:** R^2^ and *p* values (in parentheses) of linear regressions of the upper airways. In the left column, dependent variables are shown. Independent variables are shown at the top of the other columns. Only significant regressions are shown (*p* < 0.05).

	SNA	SNB	ANB	SN^GoGn	ANS-PNS	PL	PT	MPH	Age	Sex
*Nasopharynx*										
**NL**	0.043 (0.023)		0.0441 (0.021)							
**PD**	0.0758 (0.002)		0.1351 (0.001)							
*Oropharynx*										
**SPAS**						0.0509 (0.013)				
**MAS**									0.0528 (0.012)	0.0519 (0.012)
**IAS**										0.063 (0.012)
**PAS min**									0.0342 (0.043)	0.038 (0.033)
*Hypopharynx*										
**LPW**								0.0337 (0.034)		
*Soft palate*										
**PT**										
**PL**							0.1808 (1.29 × 10^−6^)			
*Hyoid bone*										
**MPH**										

**Table 6:** Reference values for the study outcomes according to published literature [21,25,26].

Class Divergence Sex	NL	PD	SPAS	MAS	PAS Min	IAS	LPW	MPH	PL	PT
Class I	/	/	13.1 ± 2.6	9.7 ± 3.1	/	12.3 ± 4.4	/	18.2 ± 4.4	36.8 ± 4	7.4 ± 1.3
Class II	/	/	14 ± 3.8	10.1 ± 3.1	/	12.9 ± 3.9	/	15.8 ± 4.8	37 ± 4	7.4 ± 1.44
Class III	/	/	12.8 ± 4.4	10.6 ± 4.5	/	13.9 ± 4.6	/	18.8 ± 5	34 ± 9.3	7.3 ± 2.1
Ipodivergence	/	/	12.9 ± 2.74	/	/	/	/	18.95 ± 4.37	34.39 ± 8.42	/
Normodivergence	/	/	12.64 ± 2.3	/	/	/	/	15.97 ± 4.97	31.07 ± 4.16	/
Iperdivergence	/	/	10.64 ± 1.83	/	/	/	/	18.74 ± 5.53	33.12 ± 3.96	/
Males	/	27.9 ± 2.5	/	10.9 ± 2.8	11.1 ± 3.2	/	19.7 ± 2.6	/	38.3 ± 1.9	11.1 ± 1.4
Female	/	25.1 ± 1.3	/	10.1 ± 2.4	10.5 ± 2.8	/	16.5 ± 3.1	/	35.6 ± 1.7	9.5 ± 1.4

## Data Availability

The data presented in this study are available on request from the corresponding author. The data are not publicly available due to the large amount of data.
